# THE UNEQUAL ENTITLEMENT TO FILIAL CARE: HOW DO OLDER CHINESE IMMIGRANTS MOBILIZE SUPPORT FROM ADULT CHILDREN

**DOI:** 10.1093/geroni/igae098.1146

**Published:** 2024-12-31

**Authors:** Xuemei Cao

**Affiliations:** Bentley University, Waltham, Massachusetts, United States

## Abstract

The broad-brush image of vulnerable older immigrants conceals their heterogeneous pathways to later life and their varied abilities to mobilize care during personal and health crises. This study examines why some older immigrants hesitate to seek help from their children whereas others feel entitled to ask for help. I conducted in-depth interviews with 42 older Chinese immigrants (aged 60 and above) in Massachusetts and ethnographic observations at various spaces where my participants congregated. Built on the cumulative advantage/ disadvantage theory, I show that international migration is a critical life transition that triggers immigrants’ unequal and gendered cumulation of material, cultural, social, and moral resources in the US and China. Those with earlier age at migration, higher socioeconomic status, and legal and social membership in the US can selectively preserve aspects of the ethnic culture of filial piety, and request help from their children as needed. By contrast, those who arrived in mid-to-late adulthood, who are of lower socioeconomic status, and who question earlier parenting and migration decisions (e.g., transnational separation) feel less entitled to seek help despite their desires for filial care. Unequal life-course resource cumulation leads to widening disparities in immigrants’ later-life health outcomes and well-being through the process of support mobilization. Considering that those who need help the most often feel least entitled to ask for help from their families, social and aging services should proactively offer support to these older adults to ensure their well-being and reduce inequalities.

